# Peculiar Virtues of Cod Liver Oil

**Published:** 1897-02

**Authors:** 


### THE PECULIAR VIRTUES OF COD-LIVER OIL.

Various explanations have been offered of the beneficial action of cod-liver oil. Some of these were fanciful enough, as for example, that which looked upon the oil as having appropriated to itself in some mysterious way the tonic virtues of the sea; others ascribed tonic properties to the contained iodine or to certain alkaloids. Other writers, again, have looked upon cod-liver oil only as an especially digestible form of fat, but they differ among themselves as to the reason of this peculiar digestibility. Buchheim believed that the presence of free fatty acids in cod-liver oil facilitated its emulsion in the intestine and thus favored absorption. On this theory a substitute for the fish oil was proposed in lipanin, or pure olive oil, to which has been added from four to six per cent. of fatty acids, the proportion found to exist in the best specimens of cod-liver oil. It was shown, however, by V. Noorden that the healthy intestine could dispose readily of neutral fat and that the addition of fatty acids was unnecessary, and Blumenfield has demonstrated that the same is true in the case of tuberculous subjects. The above mentioned experiments with sesame oil, which contains no fatty acids, would also argue against the fatty acid theory. But, whatever may be the explanation of the peculiar virtues of cod-liver oil, they are such that the remedy has hitherto, and doubtless long will hold its own against succedanea and rivals of all kinds as a builder-up and strengthener of the poorly nourished and debilitated.—*Pediatrics.*

